# Antitumor Effects of Broadleaf Vetch Against Esophageal Squamous Cell Carcinoma Through Dual Mechanisms: Suppressing EMT and Inducing Ferroptosis with Predicted Hepatorenal Toxicity—An Integrative Network Pharmacology and Toxicology Study

**DOI:** 10.3390/cancers18030370

**Published:** 2026-01-24

**Authors:** Yuxuan Xing, Siao Chen, Kang Hu, Zihan Cui, Yuhan Shao, Jingfeng Zhu, Zhimeng Chen, Jun Chen, Weijun Deng, Cheng Ding, Jun Zhao

**Affiliations:** Institute of Minimally Invasive Thoracic Cancer Therapy and Translational Research, Suzhou Medical College, Soochow University, Suzhou 215000, China; xingyx119@163.com (Y.X.); 15055615651@163.com (S.C.); wfhk04@126.com (K.H.); cuizihan0717@163.com (Z.C.); 13053660855@163.com (Y.S.); zhujingfeng0612@163.com (J.Z.); chenzhimeng1999@163.com (Z.C.); jc@suda.edu.cn (J.C.); cding@suda.edu.cn (C.D.)

**Keywords:** esophageal squamous cell carcinoma, broadleaf vetch, ferroptosis, network pharmacology, hepatotoxicity, nephrotoxicity

## Abstract

Background: Esophageal squamous cell carcinoma (ESCC) remains a highly lethal malignancy with limited effective treatments. Broadleaf Vetch (*Vicia amoena*, BV) is a traditional medicinal herb with potential anticancer properties, but its mechanisms in ESCC are not fully understood. Methods: Network pharmacology was used to identify BV-related therapeutic targets and pathways. Molecular docking validated interactions between BV components and core proteins. In vitro assays evaluated proliferation, colony formation, migration, invasion, epithelial–mesenchymal transition (EMT) markers, and ferroptosis-related indices. An ESCC xenograft model was used to assess antitumor efficacy in vivo. Results: Five major BV components and 363 ESCC-related targets were identified, highlighting the PI3K–AKT pathway and key nodes such as EGFR, AKT1, SRC, TP53, and GPX4. BV significantly inhibited ESCC cell proliferation, migration, and invasion, and reversed EMT marker expression. Ferroptosis induction was evidenced by significant Fe^2+^ accumulation, elevated reactive oxygen species (ROS) and malondialdehyde levels, alongside glutathione depletion. BV treatment also precipitated mitochondrial dysfunction. In parallel, BV downregulated GPX4 and SLC7A11. Notably, these changes were largely reversed by the ferroptosis inhibitor Ferrostatin-1. In vivo, BV suppressed tumor growth and regulated EMT- and ferroptosis-associated proteins in xenograft tissues. Conclusions: BV exerts dual antitumor effects in ESCC by suppressing EMT and inducing ferroptosis. These findings suggest BV may represent a potential multi-target phytotherapeutic candidate for ESCC.

## Simple Summary

Esophageal squamous cell carcinoma is an aggressive cancer with limited treatment options. In this study, we investigated Broadleaf Vetch (*Vicia amoena*), a traditional medicinal herb, and found that it shows antitumor effects against esophageal squamous cell carcinoma. By integrating computational network analysis with experimental verification, this study demonstrates that Broadleaf Vetch suppresses the epithelial–mesenchymal transition—a critical driver of tumor metastasis—while concurrently triggering ferroptosis, an iron-dependent form of regulated cell death characterized by oxidative stress. In animal studies, Broadleaf Vetch markedly reduced tumor growth and regulated key pathways associated with epithelial–mesenchymal transition and ferroptosis. These findings suggest Broadleaf Vetch as a potential natural multi-target candidate for the treatment of esophageal squamous cell carcinoma.

## 1. Introduction

Esophageal squamous cell carcinoma (ESCC) is the predominant histological subtype of esophageal cancer, accounting for approximately 84% of all cases [1]. It arises from the esophageal squamous epithelium and is primarily located in the proximal and middle regions of the esophagus. Globally, and particularly in China, ESCC represents nearly 90% of all esophageal cancer cases [2]. Because early symptoms are usually absent, only about 20% of patients are diagnosed at an early stage, when the five-year survival rate can reach approximately 85% [3]. In contrast, most cases are detected at advanced stages by endoscopy or chest CT, at which point the five-year survival rate falls to about 20% [4]. Despite the clinical advancements introduced by immunotherapies, specifically PD-1/PD-L1 inhibitors, therapeutic efficacy is often constrained by suboptimal response rates and a lack of reliable predictive biomarkers; consequently, post-treatment recurrence and metastasis continue to drive ESCC-associated mortality. Therefore, identifying new therapeutic targets and developing effective, low-toxicity treatment strategies are urgent challenges in ESCC research.

Broadleaf Vetch (*Vicia amoena*, BV) is a medicinal plant from the genus Vicia in the legume family. The entire herb, also known as Tougucao, has been used in traditional Chinese medicine to dispel cold and dampness, improve circulation, and relieve pain. BV is primarily distributed in northern and northeastern China and is listed in the Chinese Pharmacopoeia (2015 edition) [5] and the Dictionary of Chinese Materia Medica [6]. Clinically, it is used to treat rheumatic pain and syndromes associated with phlegm, dampness, and toxin accumulation, and it is a component of several classical prescriptions, including Kanlisha, Yangxue Rongjin Pill, and Shutong An Film [7]. More importantly, modern phytochemical studies have confirmed that plants of the genus Vicia, including the broadleaf vetch used in this study, are rich in bioactive components such as flavonoids and saponins [8,9,10,11]. Crucially, the anticancer activities of these two classes of compounds are closely linked to ESCC, the focus of this research. Beyond their antioxidant and anti-inflammatory properties, flavonoids have been directly reported in multiple studies to effectively inhibit the proliferation and invasion of ESCC cells and induce their apoptosis, with specific examples including quercetin and tricin [12,13]. Similarly, saponins have been shown to specifically inhibit the growth of ESCC cells and promote their death in both in vitro and in vivo models by regulating multiple signaling pathways [14,15]. This collective evidence suggests that the characteristic components of broadleaf vetch constitute an important material basis mediating its potential anti-ESCC activity. Related species of Vicia have exhibited antitumor activity, suggesting that this genus has potential as a source of anticancer compounds. Among them, BV shows notable pharmacological properties, making it a valuable candidate for investigating anti-ESCC efficacy and mechanisms.

Ferroptosis is an iron-dependent form of regulated cell death characterized by phospholipid peroxidation [16]. Its progression is governed by cellular processes that maintain lipid metabolism, iron homeostasis, redox balance, and mitochondrial function. Early studies focused on the SLC7A11/glutathione/GPX4 axis as a core regulatory pathway [17]. More recent evidence indicates that ferroptosis plays a crucial role in cancer biology: inducing ferroptosis can inhibit tumor growth, enhance sensitivity to chemotherapy and radiotherapy, and promote antitumor immunity, thereby offering a new therapeutic avenue. In ESCC, dysregulated GPX4 expression and abnormal glutathione metabolism have been reported [18], suggesting that targeting ferroptosis could provide novel therapeutic opportunities, particularly for reversing chemoresistance and inhibiting metastasis.

Network pharmacology—a concept first introduced by Hopkins in 2007—explores drug mechanisms by constructing complex biological interaction networks [19]. Building upon this, network toxicology, proposed by Liu in 2011 [20], integrates bioinformatics and multidimensional network analysis to elucidate interactions between exogenous compounds and biological systems and to predict potential toxicological effects. Both approaches are based on a “multi-target, multi-pathway” pharmacological model, which aligns with the holistic principles of traditional Chinese medicine (TCM).

In this study, we integrated network pharmacology, network toxicology, molecular docking, and experimental validation to comprehensively evaluate the therapeutic efficacy and safety of BV against ESCC. We constructed a multidimensional “drug–target–disease–toxicity” network to elucidate the poly-component, poly-target, and poly-pathway mechanisms underlying the antitumor effects of BV, while assessing its potential hepatorenal toxicity. This integrated approach provides mechanistic insights into the dual roles of BV in suppressing ESCC progression and maintaining safety, and supports its further development as a promising multi-target phytotherapeutic candidate.

## 2. Materials and Methods

**Figure 1:**
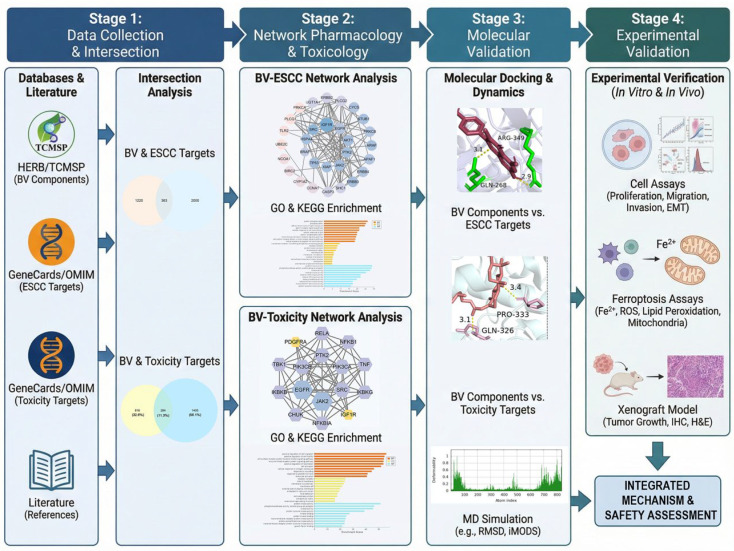
Overall workflow of the integrative network pharmacology and toxicology study of BV against ESCC.

This study integrated computational and experimental approaches to evaluate the therapeutic efficacy, potential toxicity, and molecular mechanisms of BV in ESCC. The overall workflow, combining network pharmacology, network toxicology, molecular docking, and experimental validation, is shown in Figure 1.

### 2.1. Reagents and Chemicals

BV was purchased from Shangyuantang Pharmaceutical Co., Ltd. (Nanchang, China). Quercetin, batch No. 20231028. Kaempferol and tricin were obtained from MedChemExpress (MCE, Monmouth Junction, NJ, USA). Amoenin A3 and kaempferol-3-O-α-L-rhamnoside were procured from Yuanye Bio-Technology Co., Ltd. (Shanghai, China). Antibodies against GAPDH and E-cadherin were purchased from Proteintech (Wuhan, China). Antibodies targeting N-cadherin and SLC7A11 were obtained from Cell Signaling Technology (CST, Danvers, MA, USA). GPX4 and Vimentin antibodies were supplied by Abmart (Shanghai, China).

### 2.2. Animals

Female SPF nude mice (20 ± 2 g) were obtained from the Qinglongshan Animal Center (Nanjing, China). Mice were housed under controlled conditions (22 ± 2 °C, 50 ± 10% humidity, 12 h light/dark cycle). Animals were randomly assigned to two groups (*n* = 6 per group): control group and high-dose BV group. In the pilot experiment, BV at 0.7 g/kg did not produce a measurable tumor growth inhibition under our dosing schedule; therefore, 1 g/kg was selected as the minimal dose that yielded a clear in vivo antitumor signal for subsequent mechanistic validation. BV was administered via intraperitoneal injection for 21 days. All animal procedures were approved by the Animal Research Ethics Committee of Soochow University (approval No. 202506A0690).

### 2.3. Preparation of BV Extract

**Figure A1:**
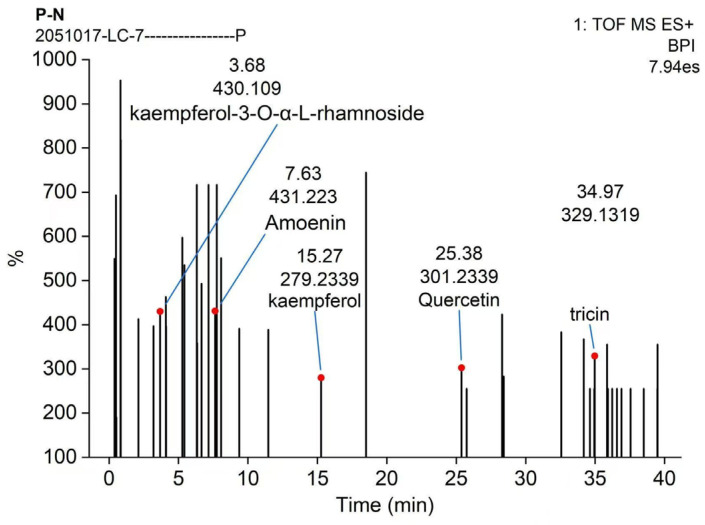
BPI chromatogram of the sample acquired by LC–TOF–MS in positive ESI mode.

A total of 2.5 g of BV powder (passed through a No. 4 sieve) was placed in a conical flask with 25 mL of 70% methanol. Ultrasonic extraction (40 kHz, 200 W) was performed twice for 30 min each. Combined extracts were filtered and evaporated to near dryness. The residue was dissolved in 25 mL of water, mixed, and extracted twice with ethyl acetate (30 mL each). Ethyl acetate layers were combined, evaporated to dryness, dissolved in methanol, transferred to a 5 mL volumetric flask, diluted to volume with methanol, and fully mixed. Extraction was verified by mass spectrometry, and the BPI chromatogram of the sample acquired by LC–TOF–MS in positive ESI mode (Figure A1, Appendix A) was used for verification.

### 2.4. Identification of Bioactive and Toxicity-Related Components

Bioactive BV components were identified using TCMSP and HERB databases. Toxicity-related components were acquired from CTD, TOXNET, and published literature [21]. To ensure data quality, only chemical-disease relationships with a CTD Inference Score > 30 were retained, a threshold recommended by CTD best practices to indicate robust, curated associations. Toxicity data were supplemented from TOXNET legacy records. Priority was given to conclusions supported by explicit experimental or clinical evidence, with their original references documented, while predictions or unvalidated annotations were treated with caution [22]. SMILES structures were obtained from PubChem and uploaded to SwissTargetPrediction and ChEMBL with “Homo sapiens” selected. All predicted targets were merged and deduplicated.

### 2.5. Acquisition of ESCC and Hepatorenal Toxicity Targets

ESCC-related and hepatorenal toxicity-related genes were retrieved from GeneCards and OMIM. Only GeneCards targets with a relevance score > 5 were retained. UniProt was used for normalization of gene symbols [23]. Venn diagrams (Origin 2024) were used to identify overlapping targets between BV and ESCC or toxicity profiles.

### 2.6. PPI Network Construction and Hub Gene Identification

Shared targets were imported into STRING (Homo sapiens, confidence score = 0.4). Networks were visualized using Cytoscape 3.8.2. The MCODE plug-in was used to identify major clusters. Hub genes were ranked using the MCC algorithm.

### 2.7. GO and KEGG Enrichment Analysis

GO and KEGG enrichment analyses were conducted using Metascape (*p* < 0.05, minimum overlap = 3, enrichment factor > 1.5; *q* < 0.05, BH correction). Results were visualized using R tools (R version 4.3.2) rom bioinformatics.com.cn. KEGG Mapper was used for pathway annotation. Herbal–compound–target–pathway–disease networks were constructed in Cytoscape 3.9.1.

### 2.8. Molecular Docking

Structures of key BV compounds were downloaded from TCMSP and PubChem, converted into MOL2 format, and energy-minimized using ChemBio3D Ultra 14.0. Protein targets (including EGFR, AKT1, SRC, TP53, and GPX4) were downloaded from the Protein Data Bank. Protein processing included removal of water molecules, addition of hydrogen atoms, and structure optimization using PyMOL 2.4.1. Docking simulations were performed in AutoDock Vina 1.1.2

### 2.9. Molecular Dynamics Simulation

Protein–ligand complexes with the highest affinity were analyzed using the IMODS server [24]. Outputs included deformability, *B*-factor, variance, covariance matrix, elastic network model, and eigenvalues using a coarse-grain model and amber94 force field [25,26].

### 2.10. Cell Lines and Culture

ESCC cell lines KYSE150 and TE-1 were purchased from ATCC (IM-H288, IM-H079, Immunoway, Plano, TX, USA). Cells were cultured in DMEM or RPMI-1640 (Gibco, Grand Island, NY, USA) supplemented with 10% FBS and maintained at 37 °C in a humidified 5% CO_2_ incubator.

### 2.11. Quantitative Real-Time PCR (qRT-PCR)

RNA was extracted using RNAzol^®^ RT reagent (GeneCopoeia, Rockville, MD, USA). cDNA was synthesized using Thermo Fisher Scientific reverse transcription kit (Thermo Fisher Scientific, Waltham, MA, USA). qRT-PCR was performed using SYBR Green (TaKaRa, Kusatsu, Shiga, Japan). The 2^−ΔΔCt^ method was used for relative quantification with GAPDH as an internal control.

### 2.12. CCK-8 Viability and IC_50_ Assay

KYSE150 and TE-1 cells (2.5 × 10^3^ cells/well) were seeded in 96-well plates and treated with BV (4 μM, 8 μM, 12 μM) [27]. After 36 h incubation at 37 °C, 8 μL CCK-8 (Beyotime, Shanghai, China) was added for 2 h. Formazan crystals were dissolved in 150 μL DMSO. Absorbance was measured at 450 nm (Multiskan^TM^ MK3, Thermo Fisher Scientific, Waltham, MA, USA). Half-maximal inhibitory concentration (IC_50_) values were calculated using GraphPad Prism 9.0.

### 2.13. EdU Cell Proliferation Assay

Cell proliferation was assessed using the BeyoClick^TM^ EdU Cell Proliferation Kit (Beyotime, Shanghai, China) with Alexa Fluor 594, following manufacturer instructions. Images were captured using a Keyence BZ-X815 fluorescence microscope (Keyence Corporation, Osaka, Japan).

### 2.14. Colony Formation Assay

Log-phase cells were seeded in 6-well plates (1000 cells/well) and treated with varying BV concentrations for 14 days. Colonies were fixed with 4% paraformaldehyde and stained with crystal violet. Colony numbers were counted and analyzed.

### 2.15. Wound Healing Assay

Cells were seeded in 6-well plates and grown to full confluence. A straight scratch was made using a 200 μL pipette tip, washed three times with PBS, and cultured in serum-free medium with BV (4 μM, 8 μM, 12 μM). Images were taken at 0 h and 48 h. Wound closure was analyzed using ImageJ (Version 2.14.0).

### 2.16. Transwell Migration and Invasion Assays

Cells in logarithmic phase were collected, resuspended at 2.5 × 10^5^ cells/mL in serum-free medium, and seeded into Transwell upper chambers. For invasion assays, chambers were precoated with Matrigel diluted at 1:4. Chambers were placed in 24-well plates containing 800 μL RPMI-1640 supplemented with 20% FBS as a chemoattractant. After incubation for 48 h at 37 °C, non-migrated cells were gently removed, and cells on the lower membrane surface were fixed with 4% paraformaldehyde and stained with crystal violet. Three random fields per membrane were imaged and quantified using ImageJ.

### 2.17. Measurement of Intracellular Fe^2+^

Cells treated with BV were washed twice with serum-free medium and incubated with FerroOrange staining solution (G1727, Servicebio, Wuhan, Hubei, China) at 37 °C for 30 min in the dark. Fluorescence was visualized using a Bioland fluorescence microscope.

### 2.18. Measurement of Malondialdehyde (MDA) Levels

Cell lysates were analyzed using a lipid peroxidation assay kit (S0131, Beyotime). Absorbance at 532 nm was measured using a Bio-Rad microplate reader (Hercules, CA, USA) to determine MDA content.

### 2.19. Glutathione (GSH) Quantification

Reduced GSH levels were quantified using a commercial GSH analysis kit (Elabscience, Elabscience, Wuhan, Hubei, China). Cell lysates were mixed with DTNB reagent and incubated in the dark for 20 min. Absorbance was measured at 412 nm.

### 2.20. Mitochondrial Membrane Potential Assay

Cells were seeded at 1 × 10^5^ cells per well in 6-well plates and treated with 12 μM BV for 48 h. After treatment, cells were stained with pre-warmed JC-1 working solution (10 μg/mL in serum-free media) at 37 °C for 20 min in the dark. Fluorescence images were captured to assess mitochondrial membrane potential.

### 2.21. Reactive Oxygen Species (ROS) Detection

Cells were loaded with 10 μM DCFH-DA dissolved in 0.9% saline and incubated at 37 °C for 30 min. Cells were washed twice, and intracellular ROS levels were quantified using a flow cytometer.

### 2.22. Ferroptosis-, Necroptosis-, and Apoptosis-Blocking Assays

To identify the dominant mode of BV-induced cell death, cells were pretreated with Necrostatin-1 (necroptosis inhibitor), Ferrostatin-1 (ferroptosis inhibitor, 5 μM), or Z-VAD-FMK (apoptosis inhibitor) before BV exposure. CCK-8 assays were performed to assess rescue effects, while flow cytometry was used to quantify cell death following 48 h of BV treatment.

### 2.23. Transmission Electron Microscopy

After BV treatment, cells were harvested, fixed, and imaged using transmission electron microscopy to assess mitochondrial ultrastructural alterations, including shrinkage, loss of cristae, and increased membrane density, characteristic of ferroptosis.

### 2.24. Western Blot Analysis

Cells or tissues were lysed using RIPA buffer supplemented with protease and phosphatase inhibitors. Protein concentrations were quantified using a BCA assay, separated via SDS-PAGE, and transferred to nitrocellulose membranes. After blocking with 5% non-fat milk, membranes were incubated with primary antibodies at 4 °C overnight, followed by HRP-conjugated secondary antibodies. Protein bands were visualized using an ECL detection system and quantified with ImageJ.

### 2.25. Xenograft Mouse Model

KYSE150 cells were injected subcutaneously into nude mice to establish ESCC xenografts. Mice were treated with BV for 21 days at concentrations of 1 g/kg. Tumor volumes were measured every three days, and tumors were harvested and weighed at the endpoint. Body weight was monitored to assess systemic tolerance. Tumor tissues were analyzed for epithelial–mesenchymal transition (EMT)-related proteins (E-cadherin, N-cadherin, Vimentin) and ferroptosis-related proteins (GPX4, SLC7A11). TUNEL assays (Roche Diagnostics, Basel, Switzerland) were used to determine apoptosis. Major organs, including the heart, liver and kidneys, were collected for H&E staining to evaluate potential toxicity.

### 2.26. Statistical Analysis

All experiments were performed in at least three independent biological replicates. Data were expressed as mean ± SD. For comparisons between two groups of continuous variables, an unpaired two-tailed Student’s *t*-test was used. For comparisons among three or more groups, one-way analysis of variance (ANOVA) was performed, followed by Tukey’s post hoc test for multiple comparisons. Gene expression differences were evaluated using the Mann–Whitney U test. Categorical variables were compared using the Chi-square or Fisher’s exact test where appropriate. Continuous variables were analyzed using an unpaired two-tailed Student’s *t*-test for two-group comparisons or one-way ANOVA for three or more groups. Statistical analyses were conducted using SPSS 22.0 and GraphPad Prism 8.0, and *p* values < 0.05 were considered statistically significant.

## 3. Results

### 3.1. Network Pharmacology and Toxicology Findings

**Figure 2:**
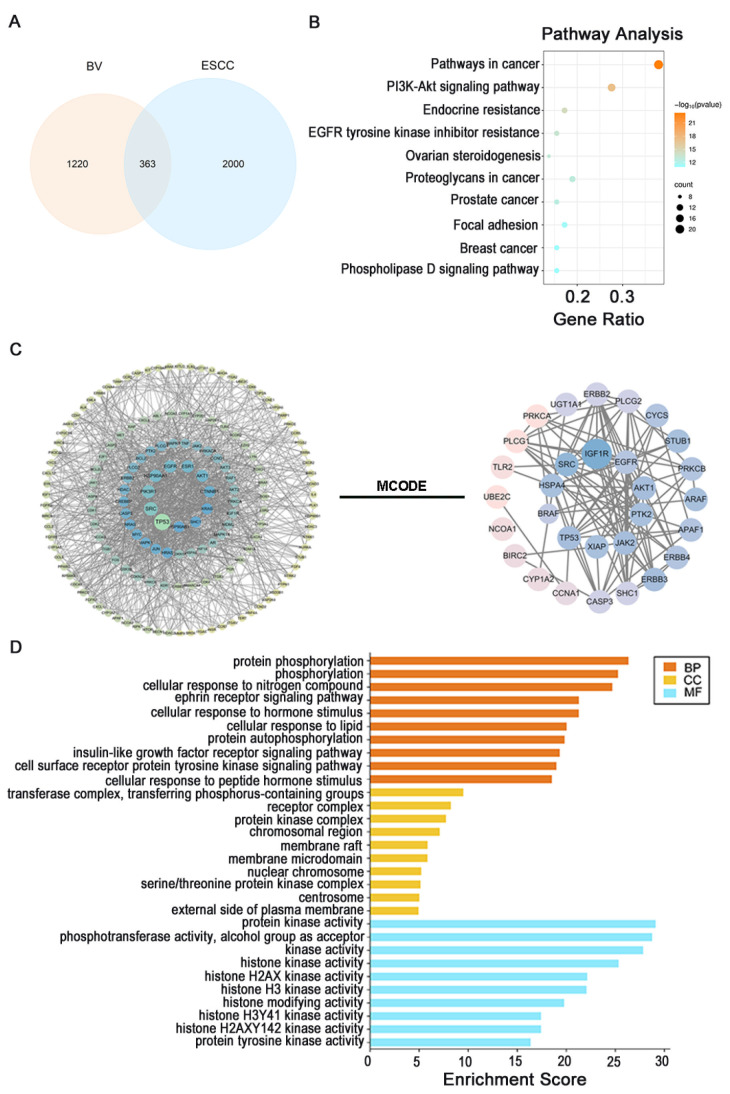
Network pharmacology analysis of the therapeutic targets of BV against ESCC: (**A**) Venn diagram showing the overlapping targets between BV therapeutic components and ESCC-related targets. (**B**) Top 10 KEGG pathways enriched for BV therapeutic targets in ESCC. (**C**) BV–compound–ESCC–target network with MCODE clustering analysis. (**D**) Top 10 GO enrichment terms (biological process, cellular component, and molecular function) for BV therapeutic targets in ESCC.

By integrating data from the TCMSP and HERB databases with literature mining and removing duplicate entries, 1583 potential targets associated with five therapeutic BV components (quercetin, kaempferol, tricin, Amoenin A3, and kaempferol-3-O-α-L-rhamnoside) were obtained. In parallel, 2363 candidate ESCC-related targets were retrieved from the GeneCards, DisGeNET, and OMIM databases. Intersection analysis identified 363 overlapping targets between BV therapeutic components and ESCC-related targets (Figure 2A).

**Figure A2:**
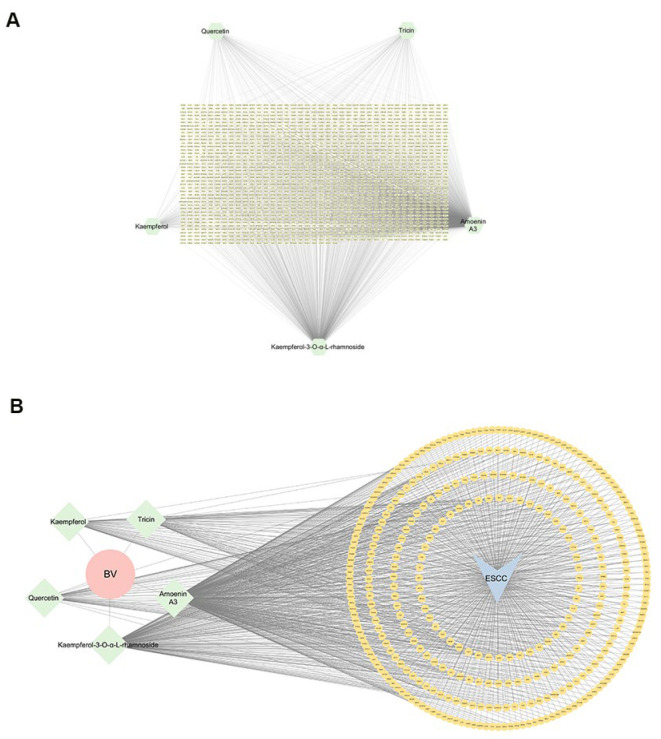
BV–compound–target network and PPI network analysis in ESCC: (**A**) BV–compound–target network illustrating the relationships between BV and its predicted targets. (**B**) PPI network of BV therapeutic targets in ESCC and the top 10 hub genes.

To further characterize the multi-component and multi-target features of BV against ESCC, a BV–compound–target network and a BV–compound–ESCC–target network were constructed in Cytoscape. In these networks, the major bioactive constituents of BV were connected with numerous ESCC-related targets, illustrating the complex interaction pattern between BV and ESCC (Figure 2C, Figure A2A, Appendix A).

The 363 overlapping ESCC-therapeutic targets were then imported into the STRING database to construct a protein–protein interaction (PPI) network. Visualization in Cytoscape yielded a network with 325 nodes and 1617 edges after removal of unconnected nodes. Degree centrality and composite scores were calculated to describe topological properties, and the MCC algorithm was applied to identify core nodes. The top 10 hub genes were TP53, AKT1, PTK2, EGFR, SRC, HSPA4, BRAF, XIAP, IGF1R, and JAK2 (Figure A2B, Appendix A).

Gene Ontology (GO) functional enrichment and Kyoto Encyclopedia of Genes and Genomes (KEGG) pathway analysis based on Metascape showed that the ESCC-related therapeutic targets of BV were significantly enriched in 846 biological processes, 43 cellular components, and 149 molecular function terms, together with 122 KEGG pathways (*p* < 0.01, *q* < 0.05). These findings indicate that BV exerts anti-ESCC effects through multiple pathways and regulatory levels. The most significantly enriched BPs were mainly related to protein phosphorylation and receptor tyrosine kinase signaling, as well as cellular responses to nitrogen compounds, hormones, lipids, and peptide hormone stimuli. The top 10 KEGG pathways included pathways in cancer, the PI3K–Akt signaling pathway, endocrine resistance, EGFR tyrosine kinase inhibitor resistance, ovarian steroidogenesis, proteoglycans in cancer, prostate cancer, focal adhesion, breast cancer, and the phospholipase D signaling pathway (Figure 2B,C). Considering both enrichment significance and disease relevance, the PI3K–Akt signaling pathway emerged as a key pathway in the anti-ESCC activity of BV (Figure 2D).

**Figure 3:**
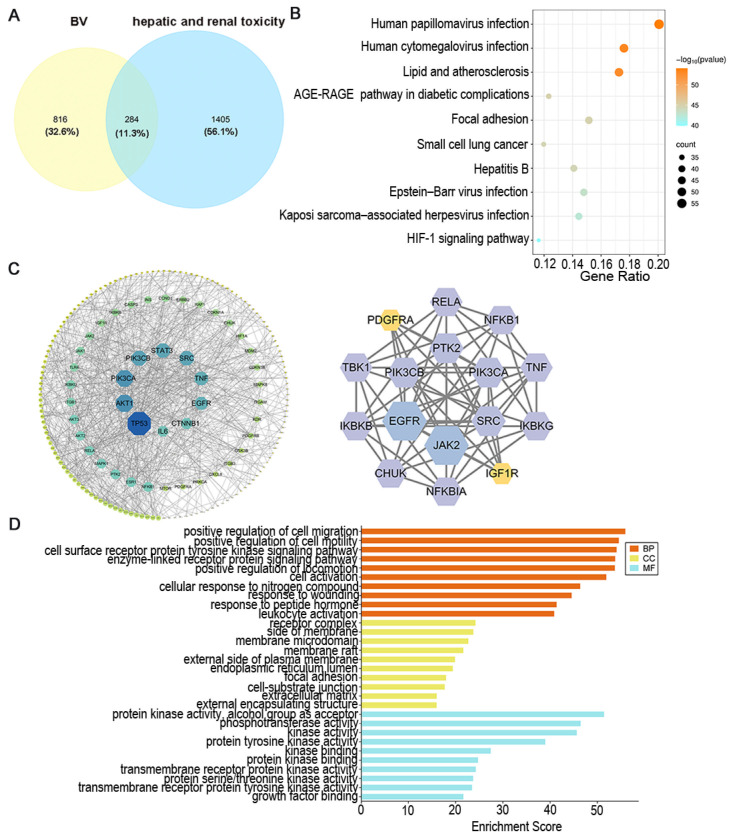
Network pharmacology analysis of BV-induced hepatorenal toxicity (HRT). (**A**) Venn diagram showing the overlapping targets between BV toxic components and hepatorenal injury–related targets. (**B**) Top 10 KEGG pathways enriched for BV toxicity–related targets. (**C**) PPI network of overlapping toxicity-related targets and the top 10 core targets. (**D**) Top 10 GO enrichment terms (biological process, cellular component, and molecular function) for BV toxicity–related targets.

For BV-induced hepatorenal toxicity, 1100 putative targets related to four toxic BV components and 1689 targets associated with drug-induced hepatorenal injury were collected from public databases. Intersection analysis revealed 284 common targets shared between BV toxic components and hepatorenal injury-related targets (Figure 3A). The PPI network constructed from these overlapping toxicity-related targets contained 185 nodes and 817 edges after exclusion of isolated nodes and low-confidence interactions, and the top 10 core targets were PTK2, JAK2, EGFR, PIK3CB, SRC, NF-κB, RELA, TBK1, TNF, and IKBKG (Figure 3C).

**Figure A3:**
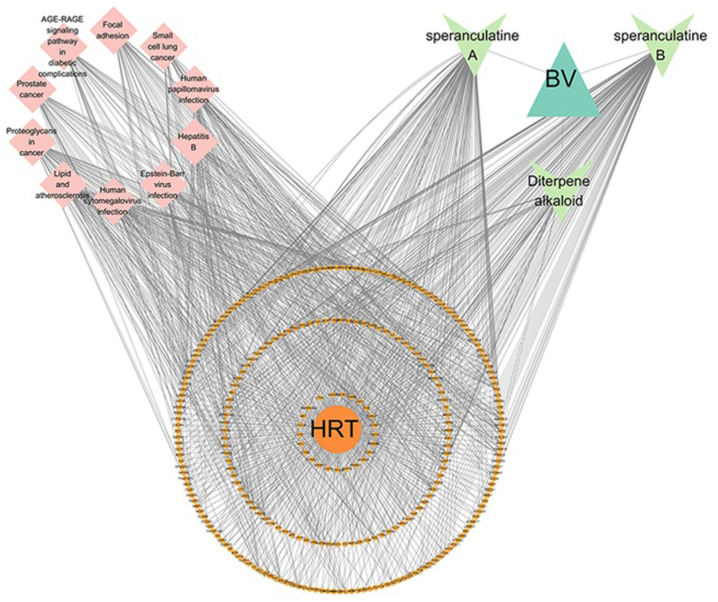
Herb–Compound–Target–Pathway–Disease (H–C–T–P–D) network depicting the regulatory mechanisms of BV-induced HRT and highlighting the principal toxic constituents.

To delineate the multi-component and multi-target mechanisms underlying BV-induced hepatorenal toxicity, an H–C–T–P–D (Herb–Compound–Target–Pathway–Disease) network was established. This network integrated BV, its toxic components, the predicted targets, the enriched pathways, and the disease node (BV-induced hepatorenal toxicity) into a single framework, within which Diterpene alkaloid, Speranculatine A, and Speranculatine B emerged as principal toxic constituents (Figure A3, Appendix A).

**Figure A4:**
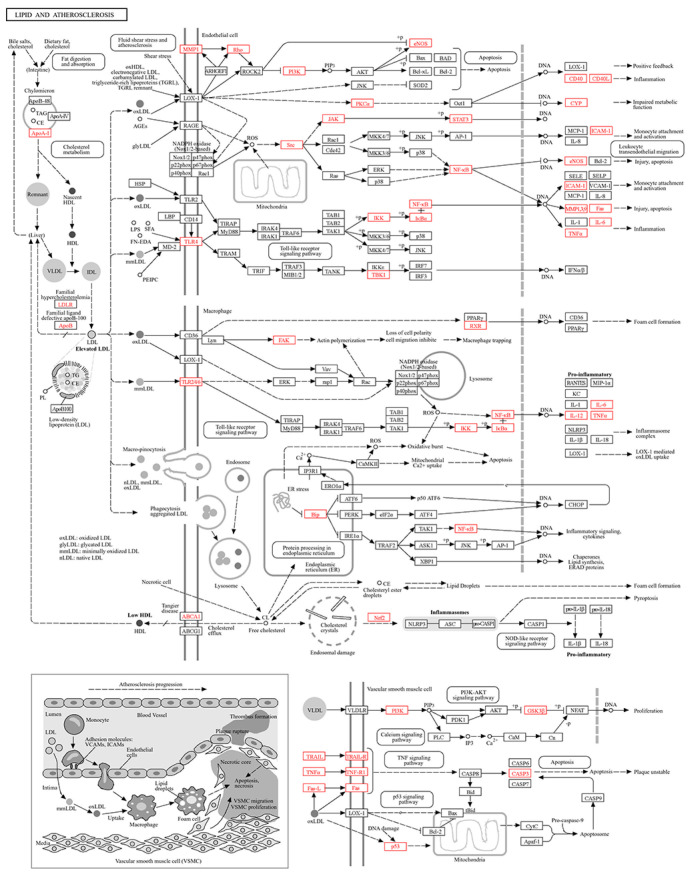
Mapping of the core “lipid and atherosclerosis” pathway using the KEGG Mapper database.

**Figure A5:**
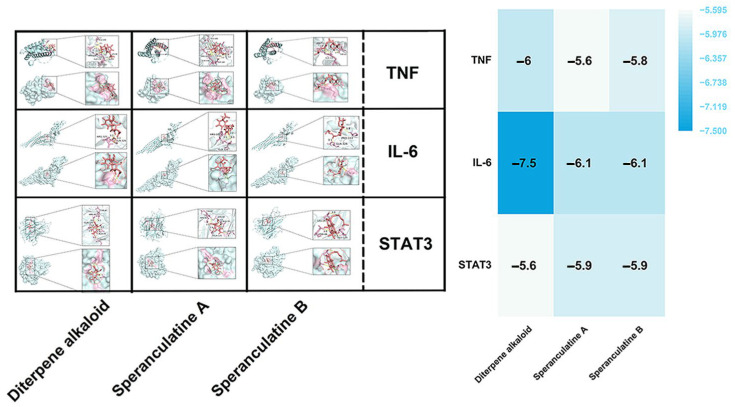
Molecular docking of core targets with key toxic BV components.

GO and KEGG enrichment analysis showed that, for BV-induced hepatorenal injury, 2648 BPs, 178 CCs, 277 MFs, and 228 KEGG pathways were significantly enriched (*p* < 0.01, *q* < 0.05). Notably, the “lipid and atherosclerosis” pathway displayed the most significant correlation with BV-associated hepatorenal toxicity. This finding implies that dysregulation of lipid metabolism and vascular inflammation may underpin the potential organ-specific injury induced by BV (Figure 3B,D, Figure A4, Appendix A). Furthermore, molecular docking of core targets with key toxic BV components supported the network pharmacology predictions and validated the predicted interactions between toxic compounds and central hepatorenal injury-related targets (Figure A5, Appendix A).

### 3.2. Molecular Docking and Dynamics Validation

**Figure 4:**
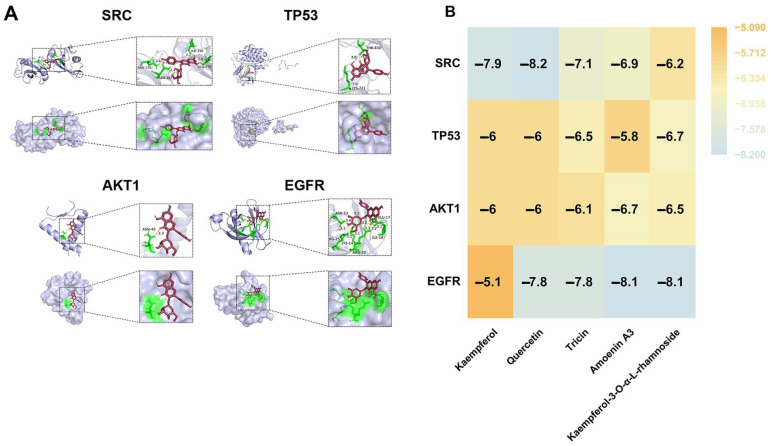
Molecular docking analysis of BV active components with four core therapeutic targets. (**A**) Three-dimensional and two-dimensional docking visualizations of Amoenin A3 and SRC, TP53, AKT1, EGFR. (**B**) Heatmap of binding energies (kcal/mol) between the five BV components and the four core targets. More negative values indicate stronger binding affinities.

To validate the interaction strength between BV and the identified core targets, the three-dimensional structures of EGFR, AKT1, SRC, and TP53 were obtained, and the 3D structures of the five major BV components were generated and energy-minimized using SYBYL-X 2.0. Molecular docking was then performed in AutoDockTools (Version 1.5.7), with these proteins as receptors and BV components as ligands. All docking pairs showed binding energies below −5.0 kcal/mol, indicating stable interactions, and several compound–target pairs had values below −7.0 kcal/mol, reflecting stronger binding affinity. The docking results revealed that, at the target level, SRC and EGFR exhibited consistently strong binding with all five BV components. At the compound level, each BV constituent demonstrated favorable binding to multiple core targets. Figure 4A presents three-dimensional and two-dimensional docking visualizations of Amoenin A3 binding with SRC, TP53, AKT1, and EGFR.

**Figure A6:**
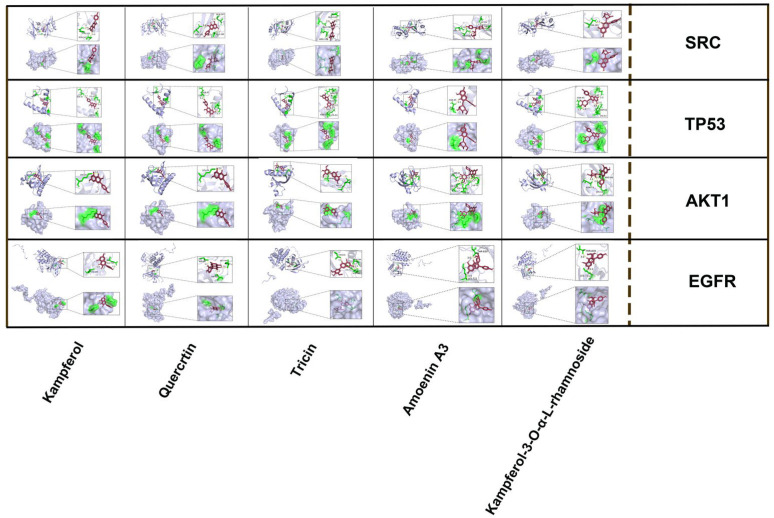
Molecular docking analysis of BV active components with four core therapeutic targets. Three-dimensional and two-dimensional docking visualizations of five principal BV components (kaempferol, quercetin, tricin, Amoenin A3, and kaempferol-3-O-α-L-rhamnoside) with four key targets (SRC, TP53, AKT1, and EGFR). For each compound–target pair, the overall protein structure, ligand-binding pocket, and predicted interaction mode are shown.

The heatmap in Figure 4B summarizes the docking results, showing that all five compounds formed stable complexes with SRC, TP53, AKT1, and EGFR. Additional detailed docking results, including the binding of other compounds with the core targets, are provided in Figure A6, Appendix A.

**Figure 5:**
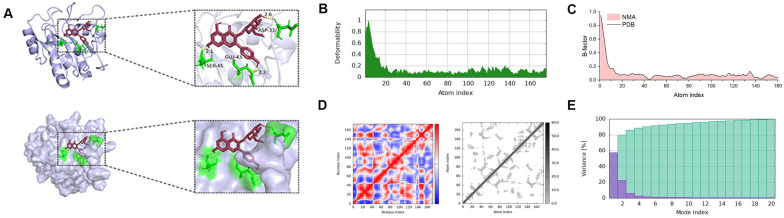
Molecular docking and molecular dynamics simulations of BV components with GPX4. (**A**) Three-dimensional and zoomed-in views of the predicted binding modes between GPX4 and Amoenin A3. (**B**) Predicted backbone deformability profiles of GPX4 in complex with Amoenin A3. (**C**) *B*-factor plots for the GPX4–Amoenin A3, comparing normal mode analysis (NMA)–predicted *B*-factors with PDB-derived *B*-factors across the residue index. (**D**) Covariance matrix and elastic network model analyses of the GPX4–Amoenin A3, showing correlated (red), anti-correlated (blue), and uncorrelated (white) motions between residues, together with the corresponding elastic network representation. (**E**) Variance associated with each normal mode for the GPX4–Amoenin A3. Bars represent the contribution of individual modes; lower overall variance and similar mode distributions indicate limited deformability and stable complex conformations. The purple region represents the protein’s global collective motions, whereas the green region represents more local, subtle atomic vibrations.

Network analysis further highlighted GPX4, a key enzyme in the ferroptosis pathway, as a potential BV target. Molecular docking demonstrated that the binding energy between GPX4 and Amoenin A3 was below −5.0 kcal/mol, indicating a stable interaction and supporting the hypothesis that BV may induce ferroptosis by targeting GPX4 (Figure 5A).

To further evaluate the stability and dynamic behavior of the GPX4–Amoenin A3 complex, molecular dynamics simulations were performed. Analysis of backbone deformability and predicted B-factors showed low flexibility in the complex (Figure 5B,C). Covariance matrix and elastic network model analyses indicated coordinated motions within the complex (Figure 5D). Additionally, the variance associated with each normal mode was low and similar among the complexes (Figure 5E), suggesting stable conformations of GPX4 upon binding to Amoenin A3.

**Figure A7:**
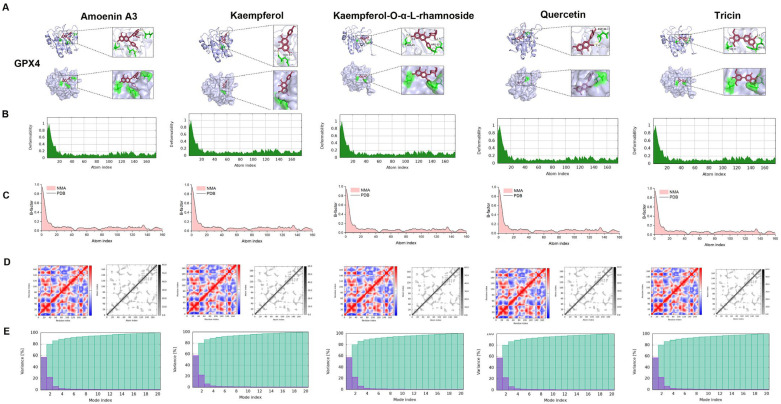
Molecular docking and molecular dynamics simulations of BV components with GPX4. (**A**) Three-dimensional and zoomed-in views of the predicted binding modes between GPX4 and five major BV components (Amoenin A3, kaempferol, kaempferol-3-O-α-L-rhamnoside, quercetin, and tricin). For each complex, the overall GPX4 structure and the ligand-binding pocket are shown. (**B**) Predicted backbone deformability profiles of GPX4 in complex with each BV component, generated by IMODS. Lower peaks indicate reduced flexibility at the corresponding residue positions. (**C**) B-factor plots for the GPX4–ligand complexes, comparing normal mode analysis (NMA)–predicted B-factors with PDB-derived B-factors across the residue index. (**D**) Covariance matrix and elastic network model analyses of the GPX4–ligand complexes, showing correlated (red), anti-correlated (blue), and uncorrelated (white) motions between residues, together with the corresponding elastic network representation. (**E**) Variance associated with each normal mode for the GPX4–ligand complexes. Bars represent the contribution of individual modes; lower overall variance and similar mode distributions indicate limited deformability and stable complex conformations. The purple region represents the protein’s global collective motions, whereas the green region represents more local, subtle atomic vibrations.

Docking results for the other BV components (kaempferol, kaempferol-3-O-α-L-rhamnoside, quercetin, and tricin) with GPX4 are provided in Figure A7, Appendix A.

### 3.3. BV Suppresses ESCC Cell Proliferation

**Figure 6:**
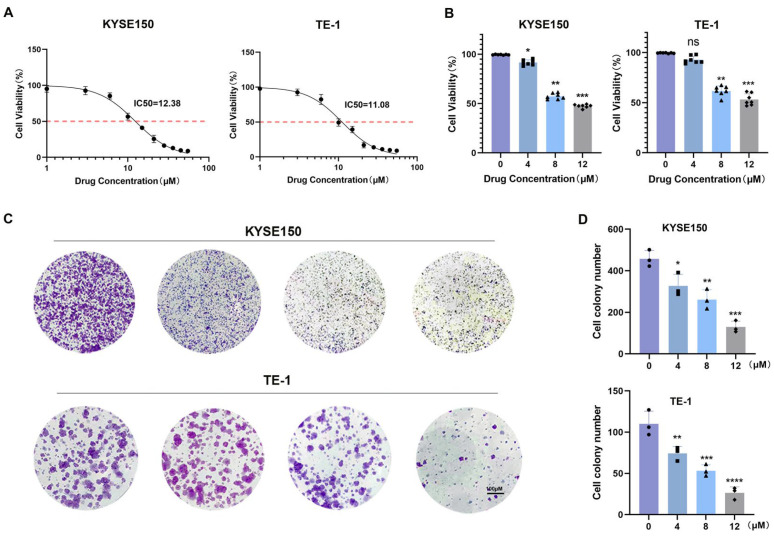
BV inhibits ESCC cell viability and proliferation in vitro. (**A**) IC_50_ values of BV in ESCC cell lines. (**B**) Cell viability of KYSE150 and TE-1 cells treated with BV for 24 h, assessed by the CCK-8 assay. (**C**) Representative images of colony formation assays in KYSE150 and TE-1 cells after BV treatment. (**D**) Quantitative analysis of colony formation. Data are shown as mean ± SD (*n* = 3). Statistical significance: ns *p* ≥ 0.05, * *p* < 0.05, ** *p* < 0.01, *** *p* < 0.001, **** *p* < 0.0001 vs. control group.

**Figure 7:**
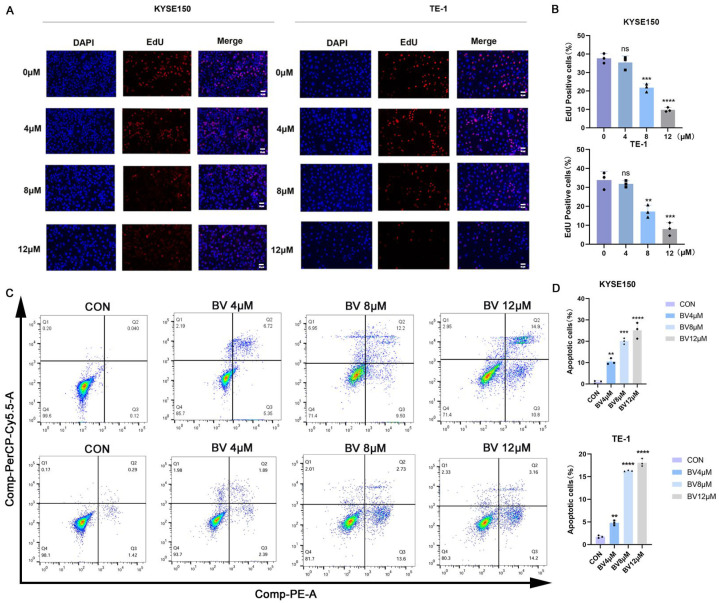
BV inhibits DNA synthesis in KYSE150 and TE-1 cells. (**A**) Representative images of EdU incorporation assays in KYSE150 and TE-1 cells following BV treatment. Scale bar = 100 μm. (**B**) Quantitative analysis of EdU-positive cells. Data are shown as mean ± SD (*n* = 3). BV induces apoptosis in KYSE150 and TE-1 cells. (**C**) Flow cytometric analysis of cell death (apoptosis) in KYSE150 and TE-1 cells following 48 h of BV treatment. (**D**) Quantitative analysis of apoptotic cells from (**A**). Data are presented as mean ± SD (*n* = 3). Statistical significance: ns *p* ≥ 0.05, ** *p* < 0.01, *** *p* < 0.001, **** *p* < 0.0001 vs. control group.

ESCC cell growth was evaluated using CCK-8 assays in KYSE150 and TE-1 cells treated with increasing concentrations of BV. BV treatment caused a dose-dependent reduction in cell viability, with significant inhibition at higher concentrations, and the IC50 values of BV in ESCC cell lines were determined (Figure 6A,B). Colony formation and EdU incorporation assays were then used to further assess the effect of BV on ESCC cell proliferation. KYSE150 and TE-1 cells were treated with 0, 4, 8, and 12 μM BV, and both assays showed that BV markedly reduced colony numbers and EdU-positive proliferating cells in a dose-dependent manner (Figure 6C,D, Figure 7A,B). In addition, flow cytometric analysis revealed that BV treatment significantly increased cell death in KYSE150 and TE-1 cells (Figure 7C,D). Overall, these data show that BV effectively inhibits ESCC cell proliferation in vitro.

### 3.4. BV Inhibits Migration, Invasion, and EMT

**Figure 8:**
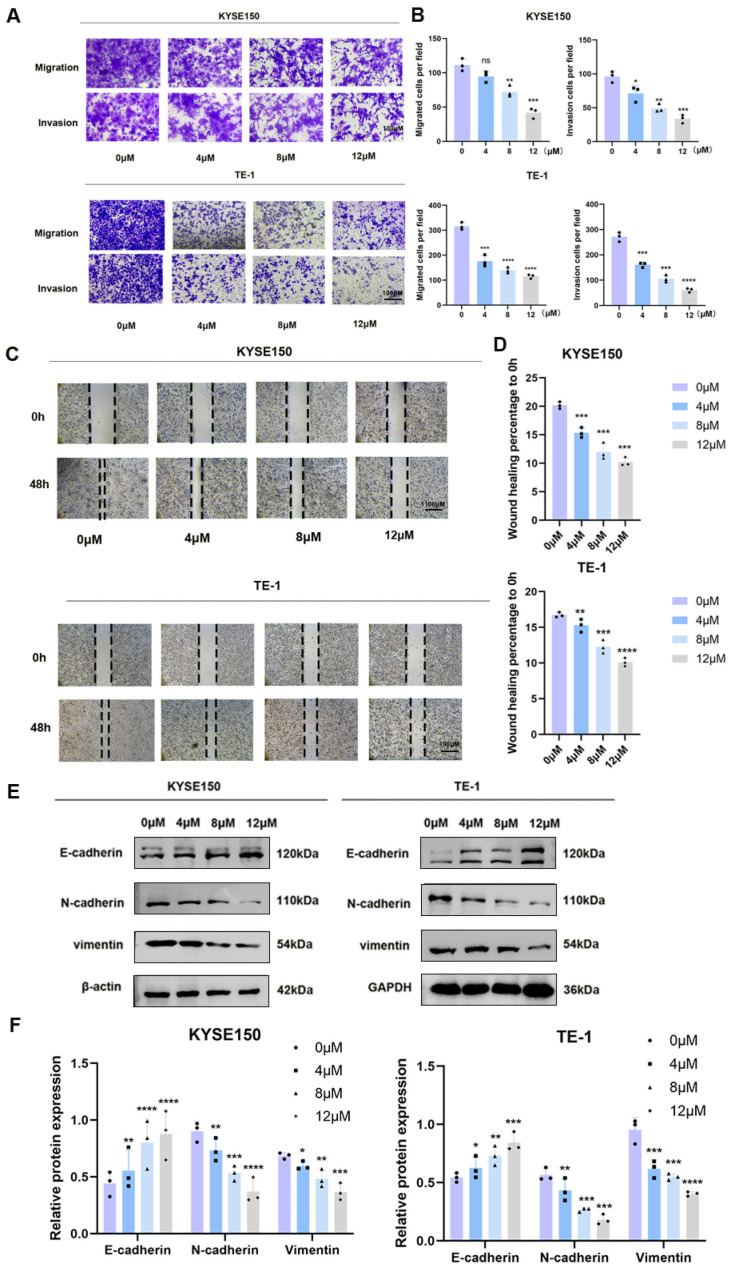
BV inhibits migration, invasion, EMT, and survival of ESCC cells. (**A**) Representative images of Transwell migration and invasion assays in KYSE150 and TE-1 cells after BV treatment (crystal violet staining; scale bar = 100 μm). (**B**) Quantitative analysis of migrated and invaded cells in Transwell assays. Data are presented as mean ± SD (*n* = 3). (**C**) Representative images of wound-healing assays showing the migratory capacity of KYSE150 and TE-1 cells after BV treatment (0 and 48 h; scale bar = 200 μm). (**D**) Quantitative assessment of wound closure. Data are expressed as a percentage of the initial wound area (mean ± SD, *n* = 3). (**E**) Western blot analysis of EMT-related proteins (E-cadherin, N-cadherin, and vimentin) in KYSE150 and TE-1 cells treated with BV for 48 h. β-actin (KYSE150) and GAPDH (TE-1) were used as loading controls. (**F**) Densitometric quantification of protein expression levels from (**E**). Data are normalized to the respective loading controls and presented as mean ± SD (*n* = 3). Statistical significance: ns *p* ≥ 0.05, * *p* < 0.05, ** *p* < 0.01, *** *p* < 0.001, **** *p* < 0.0001 vs. control group. The uncropped blots are shown in Figure S1.

Metastasis is a major cause of cancer-related mortality, so we next assessed whether BV affects ESCC cell motility and invasion. Transwell migration and invasion assays showed that BV treatment markedly reduced both the migratory and invasive capacities of KYSE150 and TE-1 cells (Figure 8A,B). Consistently, wound-healing assays demonstrated that BV slowed wound closure in a concentration-dependent manner; with increasing BV concentrations, cells exhibited progressively impaired lateral migration compared with controls (Figure 8C,D).

To determine whether these functional changes are associated with EMT, EMT-related markers were examined at the protein level. BV exposure increased the expression of the epithelial marker E-cadherin while decreasing the mesenchymal markers N-cadherin and vimentin in both KYSE150 and TE-1 cells, supporting an anti-migratory and anti-invasive role of BV in ESCC (Figure 8E,F). Collectively, these data indicate that BV not only suppresses ESCC cell motility and invasion but also reduces cell survival.

### 3.5. BV Induces Ferroptosis in ESCC Cells

**Figure 9:**
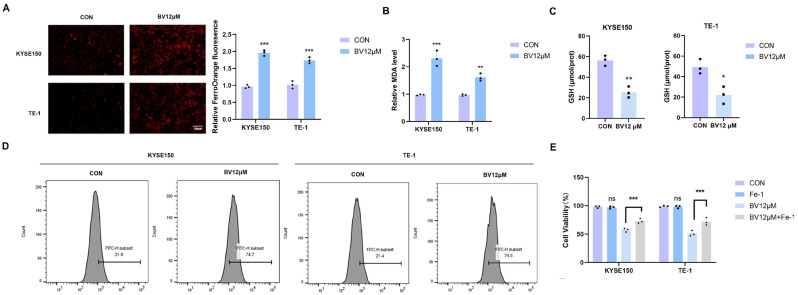
BV induces ferroptotic cell death in ESCC cells. (**A**) Intracellular Fe^2+^ levels in KYSE150 and TE-1 cells after BV treatment, measured using the FerroOrange fluorescent probe. (**B**) MDA content in control and BV-treated ESCC cells. (**C**) Reduced GSH levels in control and BV-treated ESCC cells. (**D**) Intracellular ROS levels detected using the DCFH-DA fluorescent probe. (**E**) Cell viability assessed by CCK-8 assay in KYSE150 and TE-1 cells treated with BV in the presence or absence of the ferroptosis inhibitor Ferrostatin-1 (Fer-1, 5 μM). Statistical significance: ns *p* ≥ 0.05, * *p* < 0.05, ** *p* < 0.01, *** *p* < 0.001 vs. control group.

Ferroptosis involvement in BV-induced ESCC cell death was next examined by assessing ferroptosis-related biochemical alterations. BV treatment markedly increased intracellular ferrous iron (Fe^2+^) levels in KYSE150 and TE-1 cells (Figure 9A). Moreover, BV significantly elevated MDA levels (Figure 9B) and reduced intracellular GSH content (Figure 9C). Consistently, BV exposure led to a pronounced increase in ROS production (Figure 9D), indicating enhanced oxidative stress and lipid peroxidation in ESCC cells.

**Figure 10:**
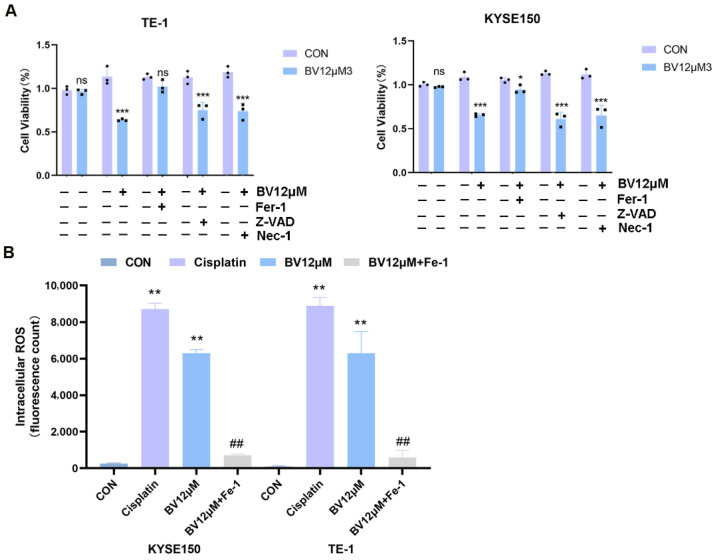
Effects of cell death inhibitors and Ferrostatin-1 on BV-induced cytotoxicity and ROS production in ESCC cells. (**A**) Cell viability in KYSE150 and TE-1 cells after co-treatment with BV and Ferrostatin-1 (Fer-1, ferroptosis inhibitor), Z-VAD-FMK (Z-VAD, apoptosis inhibitor), or Necrostatin-1. Data are shown as mean ± SD (*n* = 3). ns, *, *** indicate significant reversal of BV’s effect at *p* ≥ 0.05, <0.05 and 0.001, respectively, compared with the BV-only group. (**B**) ROS levels assessed by DCFH-DA staining in KYSE150 and TE-1 cells after the indicated treatments. Data, represented as mean fluorescence intensity, are shown as mean ± SD (*n* = 3). ** *p* < 0.01 vs. CON; ## *p* < 0.05 vs. BV.

To functionally dissect the underlying death modality, ESCC cells were pretreated with Necrostatin-1 (a necroptosis inhibitor), Ferrostatin-1 (a ferroptosis inhibitor), or Z-VAD-FMK (a pan-caspase apoptosis inhibitor) before BV exposure. Necrostatin-1 and Z-VAD-FMK provided only marginal protection against BV-induced cytotoxicity. In contrast, Ferrostatin-1 elicited the strongest protective effect (Figure 10A; Figure 9E). These differential responses indicate that ferroptosis constitutes the predominant, albeit likely not the sole, modality of cell death driven by BV. In line with this, the BV-induced increase in ROS was largely reversed by Ferrostatin-1 pretreatment (Figure 10B).

**Figure 11:**
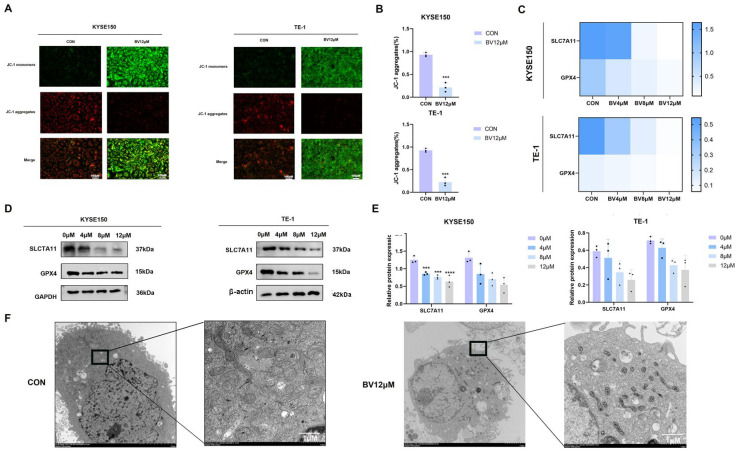
BV triggers mitochondrial dysfunction and downregulates GPX4/SLC7A11 in ESCC cells. (**A**) Representative JC-1 staining images showing mitochondrial membrane potential in control and BV-treated ESCC cells. (**B**) Quantitative analysis of the JC-1 red/green fluorescence ratio. Data are presented as mean ± SD (*n* = 3). (**C**) mRNA expression levels of GPX4 and SLC7A11 in ESCC cells treated with BV, detected by qRT-PCR. (**D**) Western blot analysis of the ferroptosis-related proteins GPX4 and SLC7A11 in control and BV-treated cells. GAPDH was used as a loading control. (**E**) Densitometric quantification of protein expression from (**D**). Data are normalized to GAPDH and presented as mean ± SD (*n* = 3). (**F**) Transmission electron microscopy images showing ultrastructural features consistent with ferroptosis, including shrunken mitochondria with reduced or absent cristae and increased membrane density in BV-treated ESCC cells. Scale bar = 2 μm. Statistical significance: *** *p* < 0.001, **** *p* < 0.0001 vs. control group. The uncropped blots are shown in Figure S2.

JC-1 staining revealed that BV treatment significantly decreased mitochondrial membrane potential in ESCC cells (Figure 11A,B), consistent with mitochondrial dysfunction commonly observed during regulated cell death. At the molecular level, qRT-PCR analysis showed that BV downregulated GPX4 and SLC7A11 mRNA expression(Figure 11C). Western blotting confirmed the corresponding decreases in GPX4 and SLC7A11 protein levels (Figure 11D,E). Both proteins are essential negative regulators of ferroptosis, and their suppression is in agreement with ferroptosis induction.

Ultrastructural examination using transmission electron microscopy provided additional morphological evidence supporting ferroptosis. BV-treated ESCC cells exhibited characteristic ferroptosis-like features, including reduced mitochondrial volume, diminished cristae, and increased membrane density (Figure 11F). Collectively, these findings support the conclusion that BV induces ferroptotic cell death in ESCC cells through Fe^2+^ accumulation, lipid peroxidation, GSH depletion, ROS elevation, mitochondrial membrane potential loss, and downregulation of GPX4 and SLC7A11.

### 3.6. BV Exhibits Anti-Tumor Activity In Vivo

**Figure 12:**
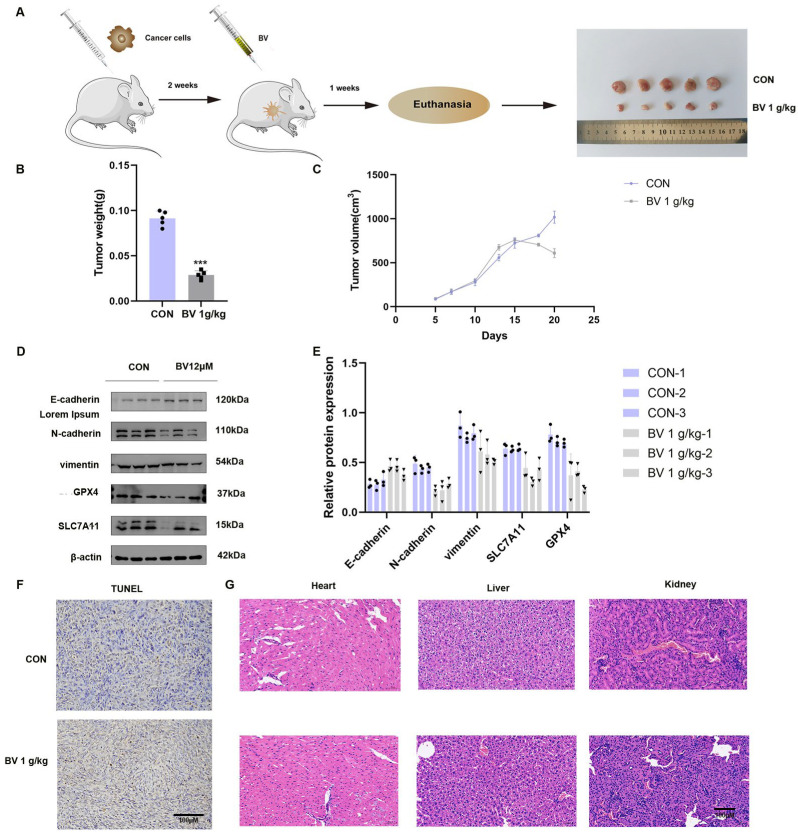
BV exerts anti-tumor effects and modulates EMT and ferroptosis in an ESCC xenograft model. (**A**) Schematic illustration of the xenograft model established by subcutaneous injection of KYSE150 cells into nude mice, followed by BV or control treatment. (**B**) Tumor growth curves recorded every 3 days during the treatment period. Data are presented as mean ± SD (*n* = 6). *** *p* < 0.001 vs. control group. (**C**) Tumor weight at the endpoint of the experiment. Data are presented as mean ± SD (*n* = 6). (**D**) Western blot analysis of EMT-related proteins (E-cadherin, N-cadherin, and vimentin) and ferroptosis-related proteins (GPX4 and SLC7A11) in tumor tissues. GAPDH was used as a loading control. (**E**) Densitometric quantification of protein expression levels from (**D**). Data are normalized to GAPDH and presented as mean ± SD (*n* = 3). (**F**) Quantitative analysis of the apoptotic rate (percentage of TUNEL-positive cells) from three random fields per sample. (**G**) Representative H&E staining images of heart, liver, and kidney tissues from each treatment group. Scale bar = 50 μm. The uncropped blots are shown in Figure S3.

**Figure A8:**
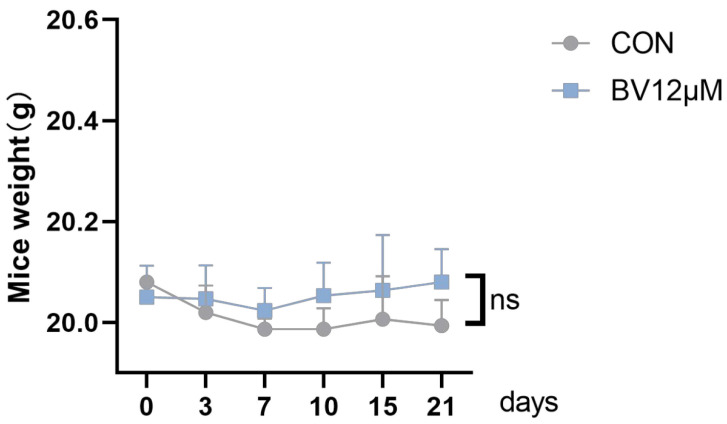
Changes in mice’s weight over time (CON vs. BV 12 μM). ns *p* ≥ 0.05.

BV antitumor activity was further evaluated in a subcutaneous xenograft mouse model established by inoculating KYSE150 cells into nude mice (Figure 12A). A 21-day BV treatment regimen (1 g/kg) significantly reduced tumor volume and tumor weight compared with the control group (Figure 12B,C). Throughout the treatment period, body weight remained unchanged (Figure A8, Appendix A), suggesting that BV was well tolerated at the tested dose.

To assess whether BV modulates ferroptosis and EMT in vivo, the expression of related proteins was examined in xenograft tumor tissues. Western blot analysis showed a marked increase in the epithelial marker E-cadherin and a pronounced decrease in the ferroptosis-associated proteins GPX4 and SLC7A11. The mesenchymal markers N-cadherin and vimentin were also significantly reduced in BV-treated tumors compared with untreated controls (Figure 12D,E). These findings are consistent with BV modulating EMT and ferroptosis-related pathways in vivo.

TUNEL staining revealed a significant increase in apoptotic cells in BV-treated tumors, further indicating enhanced tumor cell death following BV administration (Figure 12F). To evaluate the systemic safety of BV, heart, liver, and kidney tissues were subjected to H&E staining. Histopathological examination did not reveal appreciable pathological changes in any of these organs, suggesting that BV did not induce notable organ toxicity under the experimental conditions used (Figure 12G).

## 4. Discussion

BV has a long history of use in traditional medicine and has recently attracted attention because several of its major constituents, such as quercetin and kaempferol, display broad antitumor activities in multiple cancer types [28]. In this study, we provide integrated network-based and experimental evidence that BV exerts significant antitumor effects in ESCC by simultaneously suppressing EMT and inducing ferroptosis. BV inhibited ESCC cell proliferation, colony formation, migration, and invasion in vitro, and suppressed tumor growth in a xenograft model without overt systemic toxicity. These findings support the concept that BV, as a medicinal and edible resource with a favorable safety background, may represent a potential multi-target candidate for ESCC therapy.

The observed dual mechanisms are mechanistically supported by the known bioactive constituents of BV. For instance, the flavonoid quercetin, a major component of BV, has been shown to deplete intracellular glutathione and induce lipid peroxidation, leading to ferroptotic cell death in colorectal cancer [29]. Similarly, kaempferol, another prevalent flavonoid in BV, can inhibit system Xc− activity and downregulate GPX4, thereby sensitizing cancer cells to ferroptosis [30]. Additionally, saponins, another class of compounds identified in BV, have been reported to induce oxidative stress and mitochondrial dysfunction, key events in the ferroptosis cascade [31]. The coordinated downregulation of SLC7A11 (a core subunit of system Xc−) and GPX4 by BV treatment in our study aligns with the reported actions of these specific constituents, suggesting they may be key contributors to the ferroptosis induced by the whole extract.

Ferroptosis is a regulated form of cell death that tumor cells can evade through multiple adaptive mechanisms. These mechanisms include reinforcement of antioxidant defenses, tight regulation of iron and lipid metabolism, activation of pro-survival signaling pathways, and broader genetic or metabolic reprogramming [32]. Consistent with this paradigm, ESCC cells have been demonstrated to mitigate ferroptotic stress through diverse biological mechanisms, underscoring the critical contribution of ferroptosis resistance to both tumor maintenance and therapeutic recalcitrance [33]. Conversely, pharmacologic induction of ferroptosis can effectively inhibit ESCC cell proliferation and even reverse drug-resistant phenotypes [34]. Ferroptosis induction has emerged as a therapeutic strategy to inhibit ESCC growth and overcome resistance. In this study, BV induced ferroptosis in ESCC cells based on biochemical, functional, and ultrastructural evidence. Together, these findings place BV within a growing class of agents that target ferroptotic vulnerability.

Our data indicate that BV triggers ferroptosis through a coordinated disturbance of redox homeostasis and iron handling. BV treatment significantly increased intracellular Fe^2+^ levels, ROS production, MDA accumulation, and lipid peroxidation, while decreasing GSH content and mitochondrial membrane potential in ESCC cells. These biochemical alterations are consistent with the canonical definition of ferroptosis as an iron-dependent, lipid peroxidation–driven form of regulated cell death [33]. The ferroptosis inhibitor Ferrostatin-1 provided the most pronounced protection against BV-induced cytotoxicity compared with apoptosis or necroptosis inhibitors, supporting ferroptosis as a major, though not exclusive, mode of cell death in this setting. Ultrastructural analysis further revealed classical ferroptotic mitochondrial changes, including reduced mitochondrial volume, loss or diminution of cristae, and increased membrane density, which are widely recognized morphological hallmarks of ferroptosis. At the molecular level, BV markedly downregulated GPX4 and SLC7A11 expression at both the mRNA and protein levels in ESCC cells, and similar reductions were observed in xenograft tumors. Given that SLC7A11 and GPX4 are key negative regulators of ferroptosis that maintain the GSH-dependent antioxidant system, their suppression provides a mechanistic explanation for the enhanced lipid peroxidation and oxidative stress observed in BV-treated cells [32,34].

The ferroptosis-inducing activity of BV is also consistent with growing evidence that natural compounds can trigger ferroptosis by converging on the SLC7A11/GPX4 axis. For example, taraxerol, a triterpenoid derived from dandelion, induces GPX4 ubiquitination and degradation via NRF2 modulation, thereby promoting ferroptosis in breast cancer cells. Similarly, acevaltrate (ACE) from valerian perturbs iron homeostasis through dual targeting of PCBP1/2 and GPX4, leading to ferroptotic cell death. In ESCC, berbamine has been reported to enhance USP51-mediated GPX4 ubiquitination and degradation, while oridonin suppresses both SLC7A11 and GPX4, resulting in robust ferroptosis [35]. Our findings that BV decreases GPX4 and SLC7A11 and recapitulates biochemical, functional, and morphological signatures of ferroptosis place BV within this mechanistic framework and suggest that the SLC7A11/GPX4 axis is a critical downstream effector of BV action in ESCC.

Beyond ferroptosis, BV significantly inhibited ESCC cell migration and invasion and reversed EMT marker expression, as evidenced by increased E-cadherin and reduced N-cadherin and vimentin levels in vitro and in vivo. EMT is closely linked to metastatic behavior and has been associated with enhanced antioxidant capacity and resistance to ferroptosis. Our network pharmacology analysis highlighted EGFR, AKT1, and SRC as core targets of BV, and these nodes are known to participate in both EMT regulation and redox signaling in ESCC [36,37]. These data suggest that BV modulates shared signaling nodes, particularly the EGFR–AKT axis, to both attenuate EMT-associated metastatic potential and sensitize cells to ferroptosis. While we did not genetically manipulate EGFR, SLC7A11, or GPX4, the coordinated changes in EMT markers, ferroptosis-related proteins, and tumor phenotypes support a model in which BV acts on interconnected EMT–ferroptosis networks rather than isolated pathways.

From a translational standpoint, the safety profile of BV is another important consideration. Network toxicology analysis suggested that some BV components may interact with pathways implicated in hepatic and renal injury, indicating potential hepatorenal risk under certain conditions [21,38]. While our short-term in vivo study did not reveal significant histopathological damage in major organs, this finding does not preclude potential subclinical or long-term toxicity. The predicted interactions, particularly with drug-metabolizing enzymes in the liver and transporters in the kidneys, highlight the necessity for future studies to include specific serum biomarkers (e.g., ALT, AST, creatinine, BUN) and extended observation periods to fully assess organ safety.

Regarding dose selection, a lower dose (0.7 g/kg) was first evaluated in a pilot setting but did not yield a discernible antitumor effect under our regimen, whereas 1 g/kg produced a robust and reproducible tumor growth inhibition. Importantly, no overt intolerance was observed during the 21-day treatment, as reflected by stable body weight and unremarkable histology of major organs. Nevertheless, we acknowledge that a full dose–response assessment and comprehensive safety profiling (e.g., serum ALT/AST, creatinine, and BUN) are warranted in future studies to refine the therapeutic window and strengthen translational relevance.

It is crucial to acknowledge several limitations of this study that are important for interpreting our findings and guiding future research. A primary limitation of this study lies in the absence of comprehensive pharmacokinetic profiling for the BV extract. Without detailed ADME (absorption, distribution, metabolism, and excretion) data, correlating the administered dosage with clinically relevant tissue concentrations remains challenging. Second, all in vivo efficacy experiments were conducted in immunodeficient mouse models. The lack of an intact immune system prevents evaluation of how BV might interact with the tumor immune microenvironment, which is known to modulate both ferroptosis and EMT, and thus represents a significant gap in understanding its comprehensive antitumor mechanism. Finally, while we identified core targets and pathways, the precise molecular interactions between specific BV constituents (e.g., quercetin, saponins) and these targets (e.g., direct binding to SLC7A11 or GPX4) remain to be experimentally validated.

However, in our in vivo experiments, BV significantly inhibited tumor growth without causing obvious histopathological abnormalities in major organs, and body weight remained stable throughout treatment. These findings suggest that BV is well tolerated under the dosing regimen used in this study, although network predictions underscore the need for cautious evaluation of long-term exposure, higher doses, and potential cumulative toxicity.

From a translational perspective, the multi-target nature of BV is noteworthy. In this study, BV markedly inhibited tumor growth in vivo and modulated EMT- and ferroptosis-related pathways in xenograft tissues, consistent with its robust effects in ESCC cells. These findings highlight BV’s potential as a phytotherapeutic agent that may concurrently suppress metastatic traits and activate ferroptotic vulnerability. Nevertheless, several issues warrant further investigation, including the contributions of individual BV constituents, potential synergistic interactions among components, and the molecular specificity of BV–target interactions. Moreover, studies employing immunocompetent or humanized ESCC models, as well as systematic evaluation of dosing strategies and long-term administration, will be valuable for advancing the translational development of BV as a ferroptosis-activating and EMT-suppressing therapeutic candidate.

## 5. Conclusions

Collectively, this study supports BV as a multi-target therapeutic candidate for ESCC. BV suppressed tumorigenic phenotypes, including proliferation, migration, invasion, and EMT. In parallel, BV induced ferroptotic cell death characterized by iron overload, oxidative stress, and mitochondrial dysfunction, potentially via suppression of the SLC7A11/GPX4 axis. These findings underscore a dual therapeutic strategy wherein BV concurrently inhibits EMT-associated metastasis and exploits ferroptotic vulnerabilities. Further studies focusing on long-term evaluation, pharmacokinetics, and standardized preparation will be essential to advance its translational development.

## Data Availability

The datasets generated and analyzed during the current study are included within this article. Additional data are available from the corresponding author upon reasonable request.

## Supplementary Materials

The following supporting information can be downloaded at: https://www.mdpi.com/article/10.3390/cancers18030370/s1, Figure S1. Uncropped Western blot images corresponding to the blots shown in Figure 8; Figure S2. Uncropped Western blot images corresponding to the blots shown in Figure 11; Figure S3. Uncropped Western blot images corresponding to the blots shown in Figure 12.

## Abbreviations

The following abbreviations are used in this manuscript:

BV	Broadleaf Vetch
CCK-8	Cell Counting Kit-8
EMT	Epithelial–mesenchymal transition
ESCC	Esophageal squamous cell carcinoma
GO	Gene Ontology
GSH	Glutathione
HRT	Hepatic and renal toxicity
KEGG	Kyoto Encyclopedia of Genes and Genomes
PPI	Protein–protein interaction
MDA	Malondialdehyde
qRT-PCR	Quantitative real-time polymerase chain reaction
ROS	Reactive oxygen species
TCM	Traditional Chinese Medicine
WB	Western blot
