# Supplementary material for: Antitumor Effects of Broadleaf Vetch Against Esophageal Squamous Cell Carcinoma Through Dual Mechanisms: Suppressing EMT and Inducing Ferroptosis with Predicted Hepatorenal Toxicity—An Integrative Network Pharmacology and Toxicology Study
Source: Cancers (Basel). 2026 Jan 24;18(3):370. doi: 10.3390/cancers18030370 (PMC12897322; doi:10.3390/cancers18030370)
Supplement: Supplementary file 1 [file cancers-18-00370-s001.zip › cancers-4021708-supplementary.pdf]

Supplementary Materials:

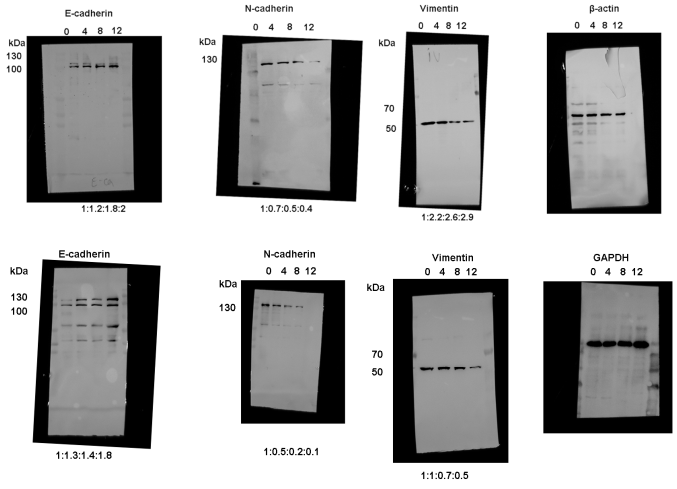

Figure S1. Uncropped Western blot images corresponding to the blots shown in Figure 8.

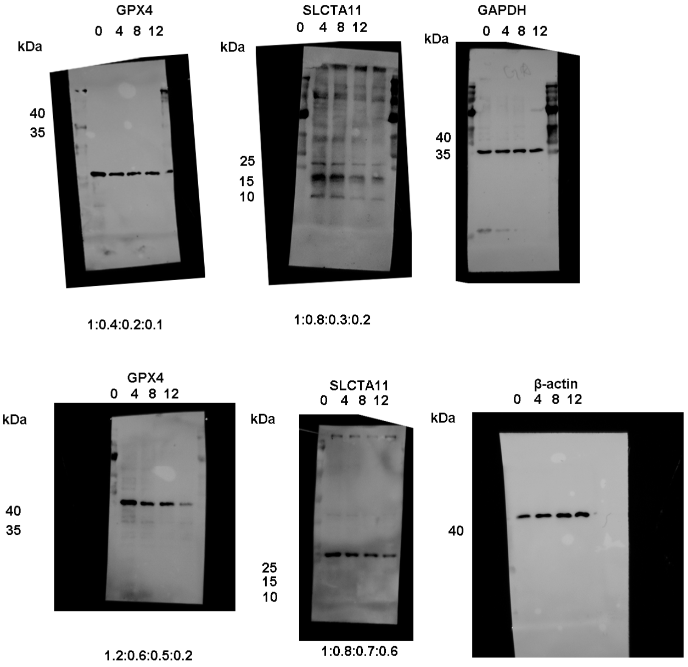

Figure S2. Uncropped Western blot images corresponding to the blots shown in Figure 11

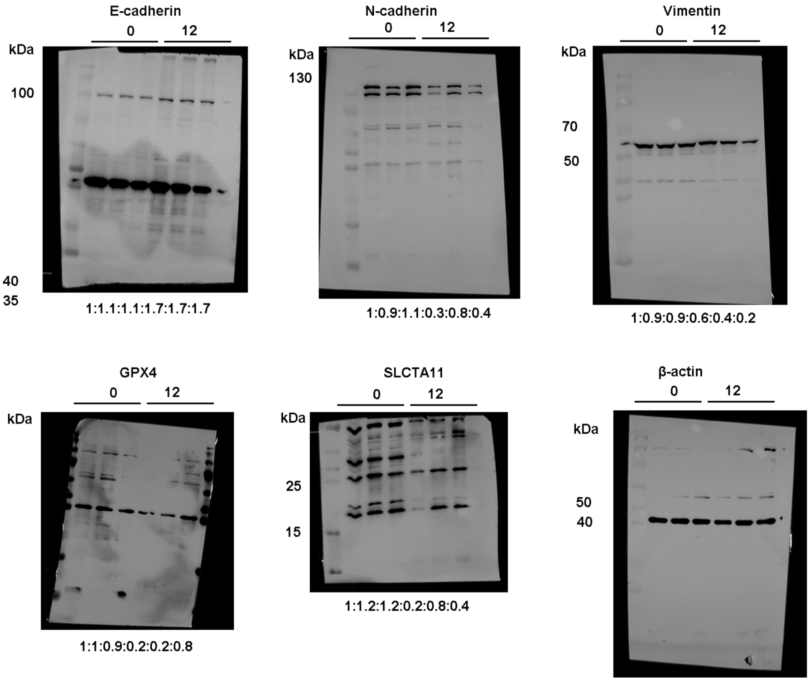

Figure S3. Uncropped Western blot images corresponding to the blots shown in Figure 12
